# MCF10CA Breast Cancer Cells Utilize Hyaluronan-Coated EV-Rich Trails for Coordinated Migration

**DOI:** 10.3389/fonc.2022.869417

**Published:** 2022-04-27

**Authors:** Niina Aaltonen, Heikki Kyykallio, Sylvain Tollis, Janne Capra, Jaana M. Hartikainen, Johanna Matilainen, Sanna Oikari, Kirsi Rilla

**Affiliations:** ^1^Institute of Biomedicine, University of Eastern Finland, Kuopio, Finland; ^2^Institute of Clinical Medicine, Clinical Pathology and Forensic Medicine, University of Eastern Finland, Kuopio, Finland

**Keywords:** hyaluronan, breast cancer, extracellular vesicle (EV), migration, filopodia

## Abstract

Invasion of tumor cells through the stroma is coordinated in response to migratory cues provided by the extracellular environment. One of the most abundant molecules in the tumor microenvironment is hyaluronan, a glycosaminoglycan known to promote many hallmarks of tumor progression, including the migratory potential of tumor cells. Strikingly, hyaluronan is also often found to coat extracellular vesicles (EVs) that originate from plasma membrane tentacles of tumor cells crucial for migration, such as filopodia, and are abundant in tumor niches. Thus, it is possible that hyaluronan and hyaluronan-coated EVs have a cooperative role in promoting migration. In this work, we compared the hyaluronan synthesis, EV secretion and migratory behavior of normal and aggressive breast cell lines from MCF10 series. Single live cell confocal imaging, electron microscopy and correlative light and electron microscopy experiments revealed that migrating tumor cells form EV-rich and hyaluronan -coated trails. These trails promote the pathfinding behavior of follower cells, which is dependent on hyaluronan. Specifically, we demonstrated that plasma membrane protrusions and EVs left behind by tumor cells during migration are strongly positive for CD9. Single cell tracking demonstrated a leader-follower behavior, which was significantly decreased upon removal of pericellular hyaluronan, indicating that hyaluronan promotes the pathfinding behavior of follower cells. Chick chorioallantoic membrane assays *in ovo* suggest that tumor cells behave similarly in 3D conditions. This study strengthens the important role of extracellular matrix production and architecture in coordinated tumor cell movements and validates the role of EVs as important components and regulators of tumor matrix. The results suggest that tumor cells can modify the extracellular niche by forming trails, which they subsequently follow coordinatively. Future studies will clarify in more detail the orchestrated role of hyaluronan, EVs and other extracellular cues in coordinated migration and pathfinding behavior of follower cells.

## Introduction

Understanding the mechanisms that drive tumor cell migration is essential in efforts for identifying strategies for effective cancer therapies. All steps within tumor progression; growth, vascularization, intravasation, extravasation, invasion, and metastasis require migration of cells. Tumor cell migration is a complex process which involves reorganization of the intracellular actin cytoskeleton and its modulators (1), components of the cell adhesion machinery (2, 3) and extracellular environment that coordinates cellular motility (4). For effective migration cells both interact with, and often modify their surrounding extracellular matrix (ECM) (5).

Hyaluronan is an abundant molecule of the tumor ECM with a fundamental role in tumor progression and regulation of migration (6). Interestingly, hyaluronan is associated with formation of filopodia (7), which are crucial in tumor cell migration *via* sensing the environment and interactions with the ECM (5). Additionally, hyaluronan induces the secretion of extracellular vesicles (EVs) (8) and accumulates on their surface, forming a thick coating on EVs (9). EVs are plasma membrane-derived particles produced by all cell types into the extracellular space and body fluids, regulating both normal physiology and pathological conditions (10). Tumor derived EVs affect the formation of tumor microenvironments and mediate cellular interactions during cancer progression (11). Lately, the contribution of EVs in tumor cell migration has received increased attention (12). Moreover, hyaluronan-coated EVs are shedding from tips of filopodia (8, 13, 14), calling for a direct testing of the role of hyaluronan and EVs in regulating the migratory capacity and invasive potential of tumor cells.

EVs have been suggested to be related to multiple aspects of cell motility, including directional sensing, cell adhesion, ECM degradation, and leader-follower behavior (12). For example, it has been shown that EV secretion is essential for promoting adhesion formation during tumor cell migration (15), and for coordination of directional cell migration (16). Coordinated cell migration relies on cellular interactions through soluble and contact-mediated signals and chemotactic gradients. During coordinated migration, leader cells facilitate the directed migration of followers, either directly by generating pulling forces *via* intercellular contacts, or indirectly by modifying the composition of the extracellular matrix (17). This kind of coordination between migrating cells is a hallmark of cancer invasion and metastasis, immune responses, angiogenesis, wound healing, and morphogenesis during embryonic development (18). All the data described above suggest an association between EVs, coordinated migration, filopodia and hyaluronan, but so far, no studies have directly demonstrated this connection.

To understand this connection in more detail, we utilized MCF10 cell series as a model of breast cancer cell migratory behavior. MCF10A cell line is a spontaneously immortalized non-malignant breast epithelial cells line that is considered normal, with no invasiveness, and no ability to form tumors in immunodeficient mice (19), while MCF10CA is the most malignant and aggressive cell line from the MCF10 series with high metastatic potential (20). We demonstrated that aggressive breast cancer cells not just produce higher numbers of EVs and more hyaluronan than normal cells, but also form trails that are coated with hyaluronan and EVs originating from cellular protrusions. Our live imaging experiments and tracking analyses in single cell level revealed that tumor cells migrate in more coordinated way than normal cells, which is attenuated when hyaluronan is enzymatically digested. We demonstrated a similar trail formation tendency of tumor cells in 3D cultures and chorioallantoic membrane (CAM) assays. The results of this study introduce a novel mechanism for hyaluronan as a guide for coordinated migration and support the role EVs as facilitators of migration.

## Materials and Methods

### Cell Culture

MCF10 human breast cell lines, MCF10A and MCF10CA, were cultured in DMEM/F12 medium (Gibco, Thermo Fischer Scientific, Waltham, MA, USA) supplemented with 5% horse serum (Invitrogen, Carlsbad, CA, USA), 2 mM glutamine (EuroClone, Pavia, Italy), 100 µg/mL streptomycin sulfate, 100 U/mL penicillin (EuroClone), 0.5 µg/mL epidermal growth factor (EGF, Sigma), 0.5 µg/mL hydrocortisone (Sigma), 0.1 µg/mL cholera toxin (Sigma), and 10 µg/mL insulin (Sigma). Both cell lines were passaged twice a week at the following split ratios (MCF10CA 1:20; MCF10A 1:25) using 0.05% trypsin (w/v) 0.02% EDTA (w/v) (Biochrom AG, Berlin,Germany). For experiments with the EV isolation, serum was purified by centrifugation at 110,000× g for 16 h and sterile-filtered with 0.22 µm syringe filters (Guangzhou Jet Bio-Filtration Co., Ltd., Guangdong, China).

### Immunostainings and Vital Stainings

The cells were cultured on 8-well Ibidi chamber slides (Ibidi GmbH, Martinsried, Germany) and fixed with 4% paraformaldehyde in PB for 20 min. The fixed cells were permeabilized for 15 min with 0.1% Triton X-100 with 1% BSA, blocked with 1% BSA for 20 min at room temperature. For detection of actin, cells were incubated for 20 min with Phalloidin-iFluor 594 Reagent (Abcam, Cambridge, UK), washed with PB, and stored at 4°C. For staining of CD44, cells were incubated overnight at 4°C with anti-CD44 antibody (1:100, Novus Biologicals, Abingdon, Oxon). After washing, the cells were incubated for 2 h with Texas red-labeled secondary antibody (l:500; Vector Laboratories Inc., Burlingame, CA, USA). For HA staining, cells were incubated overnight at 4°C with 3 µg/ml of biotinylated HA-binding complex (bHABC). After washing, the cells were incubated for 2 h with Alexa Fluor^®^ 488-streptavidin (1:500, Vector, Burlingame, CA, USA). Nuclei were labeled with 4′,6-diamidino-2-phenylindole (DAPI, 1 µg/mL, Sigma-Aldrich, St. Louis, MO, USA).

For staining of pericellular HA coat of live cells, a fluorescently labeled (Alexa Fluor^®^ 680) HA binding complex (fHABC) was used as described previously (21). Live cell cultures grown on chambered cover glasses were incubated for 2 h at 37°C with 10 µg/ml of fluorescent HABC in culture medium. CellMask™ Deep Red plasma membrane stain (1.25 µg/ml, Molecular Probes, Eugene, OR, USA) was added to the cultures immediately before imaging to label the plasma membranes. For staining of CD9 in live cultures, a FITC-labeled CD9 antibody (1:200, BioLegend, San Diego, CA, USA) was used, and nuclei were labeled with NucBlue™ (Molecular Probes) or DRAQ5™ (Biostatus Ltd., Leicesterchire, UK) DNA labels.

### Confocal Imaging

The fluorescent images were obtained with a Zeiss Axio Observer inverted microscope (40 × NA 1.3 oil objective) equipped with a Zeiss LSM 800 confocal module (Carl Zeiss Microimaging GmbH, Jena, Germany). Image processing, including three-dimensional rendering, was performed using the ZEN software (Carl Zeiss Microimaging GmbH).

### Quantitative Real-Time RT-PCR (qRT-PCR)

Total RNA from the cells was isolated using Tri Reagent (Molecular Research Center Inc., Cincinnati, OH, USA). The cDNAs were synthesized using the Verso cDNA kit (Thermo Scientific, San Jose, CA, USA). The quantitative real-time PCR was performed with Fast Start Universal SYBR Green mix (Roche Applied Science, Indianapolis, IN, USA) using the Stratagene Mx3000P real-time PCR system (Agilent Technologies, Santa Clara, CA, USA). The primer sequences were the same as used in (13). Relative mRNA expression levels were compared by using the 2−ΔΔC(T) method, with Ribosomal protein, Large, P0 (RPLP0) as reference gene.

### Hyaluronan Assay

Subconfluent cell cultures were used to measure the cellular hyaluronan secretion levels. After change of fresh medium, the cells were cultured for 48 h before the cells were counted and the media harvested for the sandwich-type hyaluronan assay as described previously (21).

### Hyaluronan Size Determination

Hyaluronan size determinations were performed using Sephacryl S-1000 (1 × 30 cm) column with 100 mM ammonium bicarbonate as a buffer. The protocol was modified from Tammi et al. (22). The column was calibrated with 2500 kDa, 500 kDa and 150 kDa hyaluronan (Hyalose, Oklahoma City, OK, USA). Cell culture medium samples in volume of 1 ml were injected into column directly (MCF10CA) or after lyophilization and dilution to volume of 1.2 ml by 100 mM ammonium bicarbonate (MCF10A). From each sample, 40 fractions (0.8 ml) were collected. Two consecutive fractions were combined and lyophilized. The dried samples were dissolved into 1% BSA-PBS and analyzed for their hyaluronan content by hyaluronan assay as described above.

### EV Isolation and Nanoparticle Tracking Analysis (NTA)

The conditioned culture media from MCF10 cells were filtered with 5 µm syringe filter (Sartorius, Goettingen, Germany) to remove cell debris. Filtered media were centrifuged at 10,000× g for 90 min at 4°C and the supernatants were centrifuged at 110,000 × g for 90 min at 4°C. Pellets from both centrifugation steps were suspended into sterile filtered PBS and combined. The size distribution and number of the EVs in isolates from MCF10 culture media were analyzed with the Nanoparticle Tracking Analyzer (Malvern Instruments Ltd., Malvern, UK) with a NS300 view unit. The following settings were used for data acquisition: camera level 13, acquisition time 30 s, and detection threshold 3. Data analysis was performed with the NTA v3.1 software (NanoSight, Amesbury, UK).

### Transmission Electron Microscopy

The EV preparations were layered onto carbon-coated glow-discharged copper grids. Grids were fixed in 2% paraformaldehyde for 10 min and contrasted using 2% neutral uranyl acetate for 10–15 min and embedded in 1.8% methylcellulose (25 Cp)/0.4% uranyl acetate. Imaging was performed with JEOL JEM 2100F transmission electron microscope (Jeol Ltd., Tokyo, Japan) operated at 200 kV.

CAM tumors were prefixed with 2.5% glutaraldehyde in 0.1 M phosphate buffer for 4h at room temperature. After an overnight wash in 0.1 M phosphate buffer, pH 7.4 and 1 h wash in H_2_O, the tumors were postfixed in 1% osmium tetraoxide and 2.22% CaCl2 in H2O and stained with 1% uranyl acetate. The tumors were dehydrated and embedded in LX-112 resin (Ladd Research Industries, Burlington, VT) and polymerized at 60°C for 48 h. The 70 nm sections were stained with 1% uranyl acetate and imaged with JEOL JEM-2100F transmission electron microscope (Jeol Ltd., Tokyo, Japan) at 200 kV.

### Correlative Light and Electron Microscopy

For correlative light and electron microscopy, cells were seeded on 13 mm cover glasses coated with Poly-D-Lysine (Sigma-Aldrich) and grown overnight. The cells were fixed and stained with CD44 antibody and bHABC probe as described above. After confocal imaging of the fluorescent stainings, cells were processed for scanning electron microscopy. Shortly, the cells were routinely dehydrated in ascending series of ethanol and hexamethyldisilazane, and finally, coated with a thin layer of gold. After processing, cells were re-localized by utilizing gridded glass bottom culture dishes and imaged with a Zeiss Sigma HD|VP (Carl Zeiss Microscopy GmbH, Oberkochen, Germany) scanning electron microscope operated at 3 kV. Adobe^®^ Photoshop was utilized to overlay of the SEM images with confocal images

### Proliferation Rate and Tracking and Analysis of Coordinated Migration

MCF10A and MCF10CA cells were seeded on the 96-well plate (2500, 3500 or 4000 cells/well). The following day, growth media was replaced with fresh growth media containing IncuCyte^®^ NucLight^®^Rapid Red Reagent (Essen BioSciences, Hertfordshire, UK) and *Streptomyces* hyaluronidase (10 TRU/ml) in selected wells. The cells were imaged every 20 min for a total of 24 h using Incucyte^®^ S3 Live-Cell Imaging System (Essen BioSciences Ltd., Hertfordshire, UK) and Incucyte S3 2021C software (Essen BioSciences, Hertfordshire, UK) was used to count the numbers of cells and the level of confluency.

### Image Analysis of Single Cell Migration

For the analysis of collective migration, MCF10A and MCF10CA cells were imaged every 20 min for 8 h, yielding a 25 time-points/frames movie for each field of view (FOV). Several FOVs were imaged for each condition, during each experiment. Cell motion was analyzed within each FOV separately as follows. First, single cell trajectories (**Figure S1A**) were extracted from the movies with the TrackMate plugin in ImageJ and computed the displacement vector of each cell along its trajectory at each time-point (See **Supplemental Material** for details). Second, for each pair of cells *i* and *j* the angle θ_i-j,k_ made by their displacement vectors was computed at each time-point *k*: θ_i-j,k_ =0 means that cells are moving exactly in the same direction at this instant of time, while θ_i-j,k_ =π means that they are moving in opposite directions. Since each FOV encompassed several hundreds of cells, hundreds of thousands of such displacement angles θ_i-j,k_ were obtained for each FOV. Third, the distribution of the displacement angles was computed, as illustrated for two typical FOVs of normal cells (**Figure S1B**, top) and cancer cells (**Figure S1B**, bottom). From this distribution, the correlation index (CI) was computed for each FOV as the ratio between the peak (around θ=0) and the basal (around θ=π) occurrence levels (**Supplemental Material** and **Figure S1B**). Therefore, equally distributed displacements (100% uncorrelated motion, flat distribution) yield CI~1, and strongly correlated collective motion corresponds to a majority of colinear displacements θ_i-j,k_~0 and large CI values. This analysis was performed in Matlab R2019b (the Mathworks) using custom dedicated scripts.

### Data Processing and Statistics

As illustrated on **Figure S1B** the CI as defined above was larger for cancer cells compared to normal cells and for untreated cancer cells compared to hyaluronan-degrading enzyme-treated cancer cells in most FOVs, and across all replicate experiments. However, we observed differences in the scaling of the CI across different replicate experiments, which could stem from different cell densities or culture conditions across experiments. Hence, to be able to aggregate data from multiple replicates without artefactually increasing data variance, we normalized the CI of each FOV of each experiment to the median CI across all control FOVs of the same experiment (normal cells for **Figure 5A**, or untreated cancer cells for **Figure 6G**), yielding the normalized CI shown on those Figure panels.

To assess the statistical significances of the differences in median CI across conditions, we performed Wilcoxon rank tests using Matlab’s ranksum function. The differences in CI for cancer versus normal cells and treated versus untreated cancer cells were statistically significant with p-values close to 0.01. To further demonstrate that such p-values were unlikely to originate by chance from the variability in our data coupled with a limited number of FOVs, we randomized the CI values for sample and control FOVs and repeated the Wilcoxon ranksum tests for 100 randomization trials. P-values lower than 0.05 were obtained in only 2-3% of the trials, limiting to this extent the odds that our conclusions arise from sample-to-sample variability and limited sampling (limited number of FOVs).

### Chick Chorioallantoic Membrane (CAM) Assays

Fertilized white Leghorn chicken eggs were incubated at 37°C under constant humidity, starting at embryo development day 0 (EDD0). Separation of the CAM was induced on EDD4 by piercing the eggshell. On EDD8 cells were collected, suspended in PBS, and Corning^®^ Matrigel^®^ Matrix GFR Phenol Red Free (Thermo Fisher Scientific Inc., Göteborg, Sweden) (1:1), and implanted on the CAM (10^6^ cells per egg). On EDD13, the tumors were photographed *in ovo* and excised. Tumor area was measured on photographs from 8-10 eggs per cell line.

Tumors were fixed in 3% paraformaldehyde, embedded in paraffin and cut in 5 µm sections. The sections were deparaffinized and rehydrated with routine protocols (xylene for 2 × 5 min, absolute EtOH for 2 × 2 min, 94% EtOH for 2 × 2 min), and washed with dH2O for 20 s. The deparaffinized sections were subjected to antigen retrieval by incubation in 10 mM citrate buffer, pH 6.0 for 15 min in a pressure cooker at 120°C. To block endogenous peroxidase, the sections were treated for 5 min with 1% H2O2. After washing with 0.1 M Na-phosphate buffer, pH 7.4 (PB), the sections were incubated in 1% bovine serum albumin (BSA) in PB for 30 min to block nonspecific binding. For hyaluronan staining, sections were incubated overnight with biotinylated complex of HA-binding region of bovine articular cartilage aggrecan G1 domain and link protein (bHABC) diluted in 1% BSA. The intensity of HA staining was quantified using the color deconvolution algorithm for DAB in ImageJ and the optical density was calculated with the formula log (max intensity/mean intensity). For staining of mitotic cells, sections were incubated overnight with primary antibody against proliferation marker protein Ki-67 (Dako, Glostrup, Denmark) and after washing, for 1 h with biotinylated antimouse secondary antibody (1:1000, Vector Laboratories). Stainings were visualized with the avidin–biotin peroxidase method (Vectastain Kit, Vector Laboratories) followed by incubation for 5 min in 0.05% diaminobenzidine (Sigma) and 0.03% hydrogen peroxide in PB, yielding a brown reaction product. The nuclei were stained with Mayer’s hematoxylin. Stained sections were imaged with Zeiss Axio Imager M2 light microscope (Carl Zeiss Microimaging GmbH, Zeiss, Jena, Germany). Stained sections were scanned by Nanozoomer XR digital slide scanner (Hamamatsu Photonics K.K., Hamamatsu City, Japan) at 20× and evaluated by the automated Oncotopix image analysis software v2018.2 (VisioPharm, Hoersholm, Denmark) provided by the Biobank of Eastern Finland.

For fluorescent stainings, the deparaffinized CAM tumor sections were treated with 50 mM glycine for 20 min at room temperature to quench any autofluorescence. The sections were blocked with 1% bovine serum albumin for 30 min, followed by an overnight incubation at 4°C with the primary antibodies against CD44 (Novus Biologicals). After washing, the sections were incubated for 1 h with the secondary antibodies (1:1000, Texas Red anti-rabbit IgG, Vector and 1:1000, Dylight 488-streptavidin, Vector). Nuclei were labelled with DAPI (1 μg/ml, Sigma-Aldrich). The sections were mounted in Vectashield (Vector H-1000, Vector) and the samples were imaged with confocal microscope.

### Statistical Analyses

Statistical analyses were carried out using the GraphPad Prism version 5.00 for Windows (Graph-Pad Software, San Diego, CA, USA). The significance of differences between groups was tested using Mann–Whitney test or Student’s t-test. Differences were considered significant when p < 0.05.

## Results

### MCF10CA Tumor Cells Produce Higher Levels of Hyaluronan and Have Increased HAS3 Expression Levels as Compared to MCF10A Normal Breast Epithelial Cells

**Figure 1:**
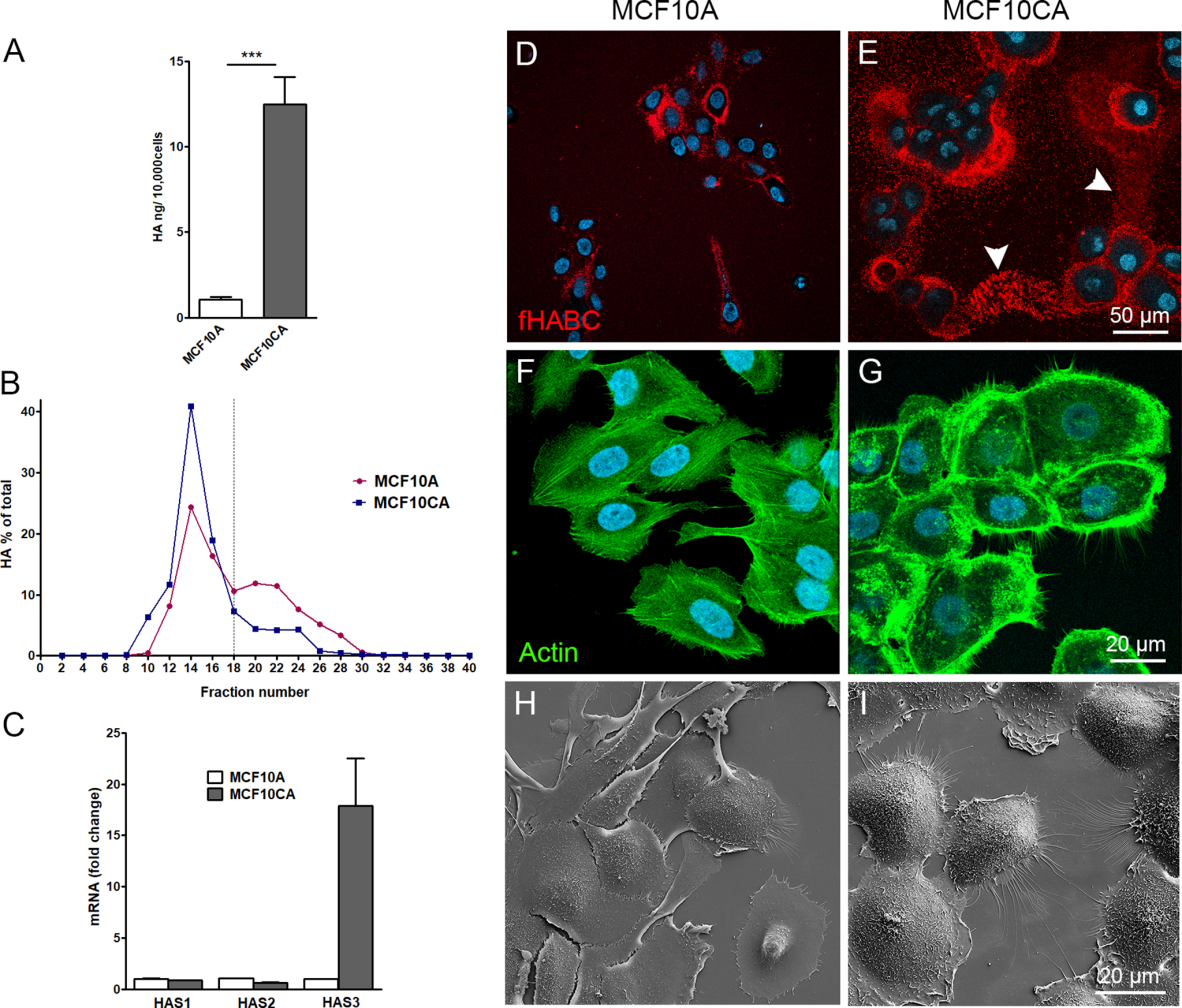
MCF10CA cells synthesize more hyaluronan and express higher levels of HAS3 than MCF10A cells. Hyaluronan secretion levels by MCF10A and MCF10CA cell lines analyzed by hyaluronan assay **(A)** and analysis of relative molecular weight distribution of produced hyaluronan **(B)**. Relative expression levels of HAS isoenzymes **(C)**. Confocal images of live MCF10A **(D)** and MCF10CA **(E)** cells stained with fHABC to detect pericellular hyaluronan (arrowheads in E indicate HA-rich trails) and fixed cell stained with phalloidin to show actin cytoskeleton of MCF10A **(F)** and MCF10CA **(G)**. Scanning electron microscopic images of MCF10A **(H)** and MCF10CA **(I)**. Blue = nuclei in **(D–G)**. The data represent means ± SE of 6 independent experiments in **(A)** ± SE of 3 independent experiments in **(B, C)** ***p < 0.001, Mann–Whitney test.

To compare the hyaluronan production activity of non-malignant MCF10A breast epithelial cells and malignant MCF10CA cells, hyaluronan secretion levels in the culture media were analyzed by hyaluronan assay and size analysis. MCF10CA cells secreted significantly higher levels of hyaluronan than MCF10A cells (**Figure 1A**), with an increased fraction of high molecular weight hyaluronan (85.5% and 65.9%, was high molecular weight in MCF10CA, and MCF10A, respectively) than normal MCF10A cells (**Figure 1B**). To find out, which of the three isoforms of hyaluronan synthases are mainly responsible for this increase, we analyzed the relative expression levels of HAS isoenzymes in both cell lines by qPCR. Both cell lines expressed all HAS isoenzymes, but the expression level of HAS3 was clearly higher in MCF10CA cells as compared to the levels of MCF10A cells (**Figure 1C**). Localization of pericellular hyaluronan was studied in live cells with fHABC hyaluronan binding probe. MCF10CA cells formed larger hyaluronan coats around them than MCF10A cells (**Figures 1D, E**). Interestingly, hyaluronan seemed to form trails on the substratum between individual MCF10CA cells (arrowheads in **Figure 1E**). We next studied the morphology of fixed, phalloidin-stained cells. We found that the cytoskeletal organization differed between the two cell lines, with denser actin peripheral accumulation and higher number of filopodia in MCF10CA cells compared to MCF10A cells (**Figures 1F, G**), in agreement with previous findings (23). Higher number of both lateral and dorsal filopodia in MCF10CA cells was confirmed by scanning electron microscopy that reveals the cells’ surface morphology (**Figures 1H, I**).

### Tumor Cells Secrete More EVs Than Normal Cells and Form Trails Rich in Hyaluronan and Hyaluronan-Coated EVs

**Figure 2:**
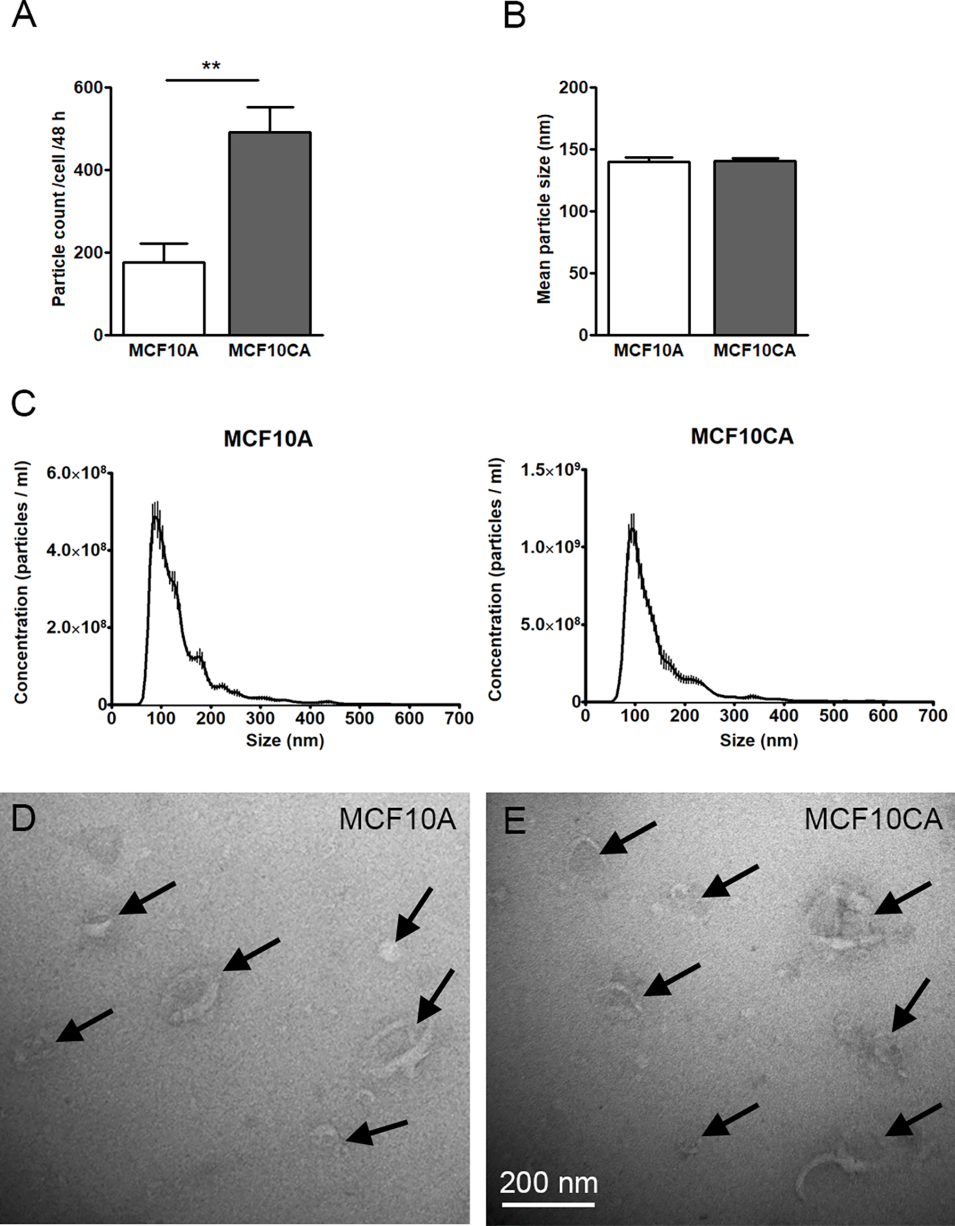
MCF10CA cell produce more EVs than MCF10A cells. Particle counts **(A)**, mean particle sizes **(B)** and size distributions **(C)** of EV isolates from MCF10 cell lines analyzed by NTA. TEM images from the same isolates are shown in **(D, E)**. The data represent means ± SE of 5-6 independent experiments in **(A, B)** and means ± SE of 5-6 independent experiments in **(C)** **p < 0.01, Mann–Whitney test. Arrows point EVs in panels **(D, E)**.

We next sought to compare the EV production activity of the two MCF10 cell lines. In this purpose, we first isolated EVs from culture media and performed NTA analysis where isolated vesicles were counted and sized. We found that MCF10CA cells produced significantly more EVs (about 2.5-fold, **Figure 2A**), but of similar size (**Figures 2B, C**), than MCF10A cells. Likewise, both cell lines produced typical cup-shaped EVs with similar morphology as revealed by transmission electron microscopy (arrows in **Figures 2D, E**). Hence, the number - but not the size or shape - of EVs was upregulated in cancer cells.

**Figure 3:**
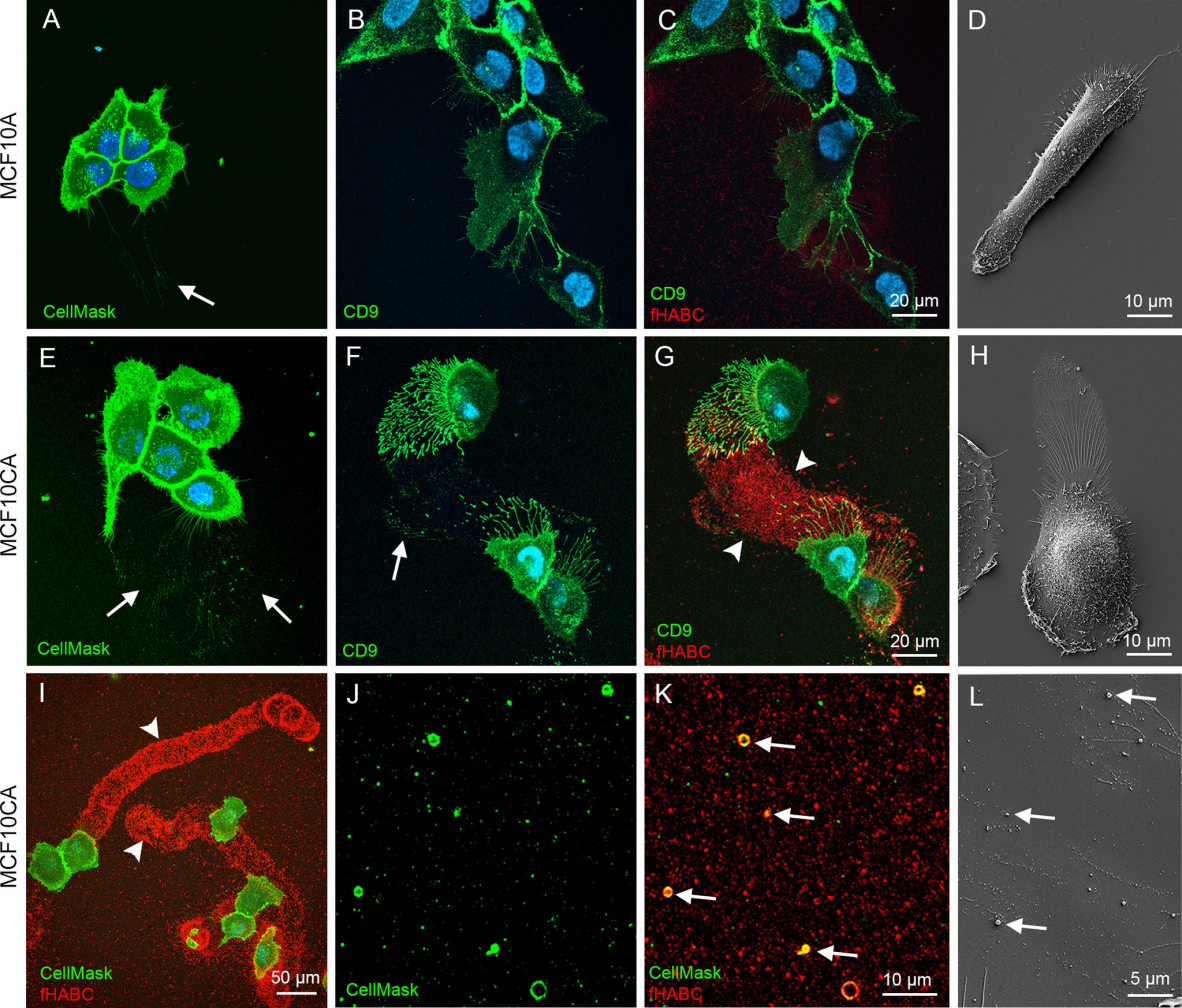
MCF10CA cells form hyaluronan-rich trails that contain plasma membrane-derived vesicles. Live MCF10 cells stained with CellMask^®^ to label lipid membranes **(A, E)** and FITC-conjugated antibody against EV marker CD9 **(B, F)** co-stained with fHABC to visualize hyaluronan **(C, G)**. A lower-magnification image **(G)** of double-staining with CellMask and fHABC shows the trails of up to several hundreds of micrometers long coated with hyaluronan. Scanning electron microscopic images of MCF10A **(D)** and MCF10CA **(H)**. Panel **(I)** shows a lower magnification overview of trails of MCF10CA cells. A higher-magnification images of the trail-areas show HA-coated (red) EVs (green) of different diameter **(J, K)**. A higher magnification scanning electron microscopic image from the trail area **(L)**. Arrows in all panels point membrane-derived vesicles and arrowheads show hyaluronan-rich trails.

We next aimed to confirm these findings in live MCF10 cells. In this purpose, MCF10A and MCF10CA subconfluent monolayer cultures were stained *in situ* with the CellMask^®^ plasma membrane marker (that also labeled EVs), FITC-labeled antibody for the EV-marker CD9, and the fHABC probe to detect hyaluronan. In agreement with our fixed cells data (**Figures 1D, E, H, I**), MFC10A cells exhibited substantially less filopodia than MCF10CA (**Figures 3A–D**, **E–H** respectively). EVs seemed to originate from those plasma membrane protrusions (arrows in **Figures 3A, E**). Some EVs were also detected on the bottom of the plate (arrow in **Figure 3A**), in particular in MCF10CA cancer cells where a high number plate-adherent EVs were arranged as trails next to cells with high number of long plasma membrane protrusions (arrows in **Figure 3E**). Double staining with CD9 and fHABC (**Figures 3B, C, F, G**) showed that the filopodia and EVs were strongly positive for CD9, and that the trails generated by MCF10CA cells were hyaluronan-rich and localized in the same areas than CD9-positive protrusions and EVs (**Figures 3F, G**). These structures were all less pronounced in MCF10A cells, with some filopodia, EVs and a weak hyaluronan staining (**Figures 3B, C**). Scanning electron microscopy corroborated fluorescence imaging findings, showing a lower number of protrusions and EVs in MCF10A cell cultures (**Figure 3D**), as compared to MCF10CA cells with clear trails of filopodia and EVs (**Figure 3H**). A lower magnification overview of MCF10CA cells labeled with CellMask (pseudo colored green) and fHABC (red) revealed the huge length of hyaluronan-rich trails, reaching up to several hundreds of micrometers (**Figure 3I**). A higher magnification image from trail area of the same live MCF10CA cell culture shows EVs of variable size (**Figure 3J**), most of which carried hyaluronan (arrows in **Figure 3K**). In addition to EV-associated hyaluronan, also “free” hyaluronan was detected in trail areas (**Figure 3K**). A higher magnification scanning electron microscopic image from the trail area revealed single EVs of variable size (arrows in **Figure 3L**). Hence, tumor cells generate hyaluronan-, EV- and hyaluronan-coated EV-rich trails on their neighbor substrate.

**Figure 4:**
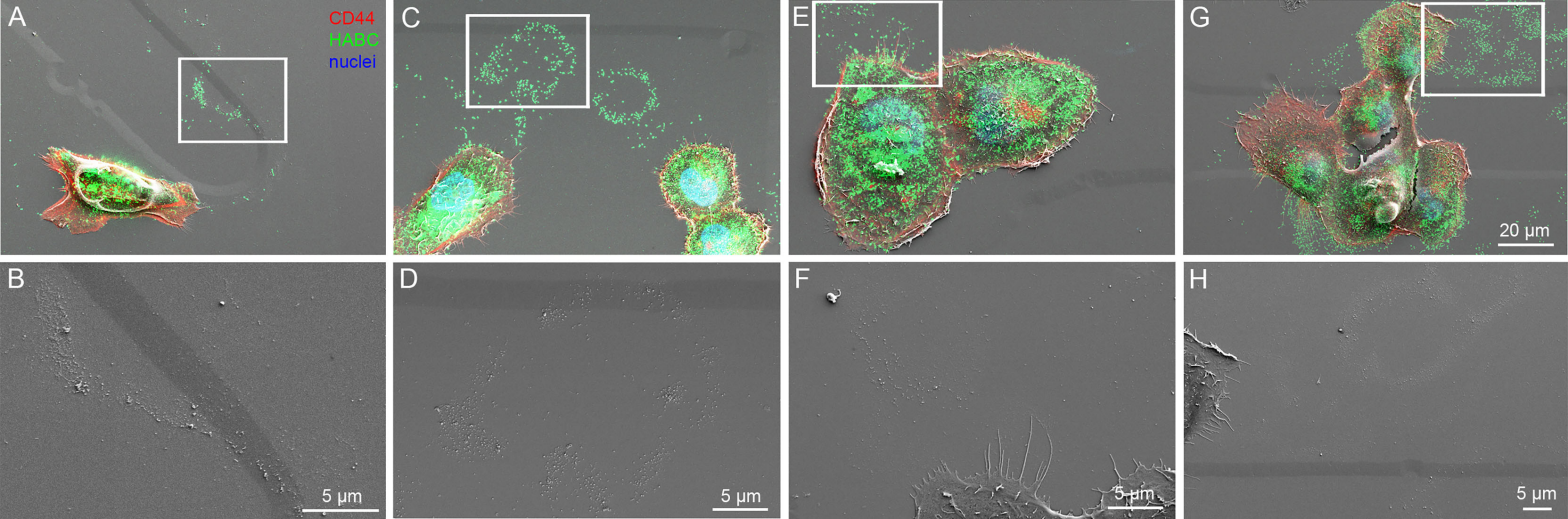
Visualization of HA-positive trails with correlative light and electron microscopy (CLEM). Single cells or groups of cells are presented as overlay images of confocal 3D projections from CD44 and fHABC stainings and scanning electron microscopy **(A, C, E, G)** and high magnification SEM images from the selected areas indicated by white boxes are shown in **(B, D, F, H)**.

Next, we aimed to visualize the ultrastructure of hyaluronan-rich trails formed by MCF10CA cells in more detail with correlative light and electron microscopy (CLEM). The fluorescence staining with CD44 antibody and bHABC probe to detect hyaluronan indicated that fixation decreased the number and length of filopodia as compared to live cells (**Figure 3**), as previously shown (23). Intensity of hyaluronan staining on trails was also diminished in fixed cells, but clearly visible trails with hyaluronan positivity were detected (**Figures 4A, C, E, G**). Correlation of the SEM and fluorescence images and higher resolution imaging from selected areas (white boxes in 4A, C, E, and G) indicated that the hyaluronan-rich trails contained EVs and also other ECM material that was adhered to the bottom of the plate and left as “slime trails” behind migrating cells, (**Figures 4B, D, F, H**).

### Tumor Cells Migrate in a More Coordinated Way Than Normal Cells

**Figure 5:**
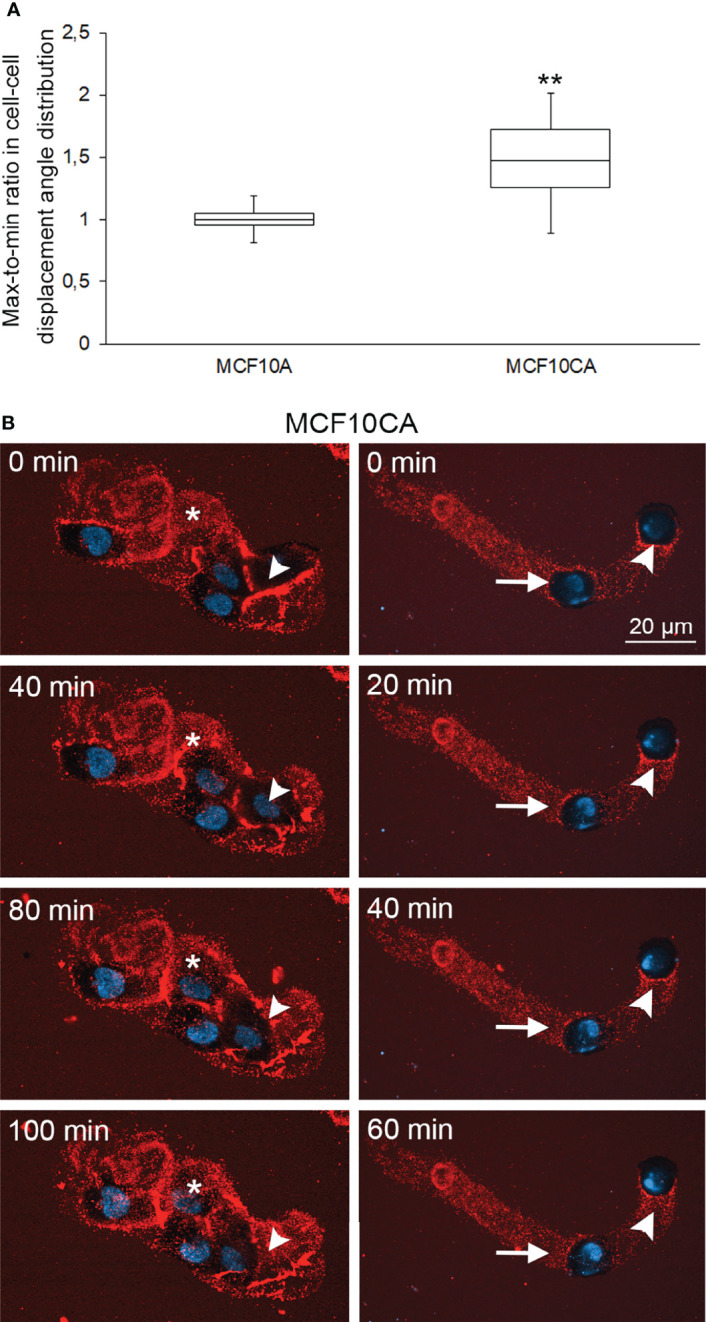
Collective cell motion is more coordinated in cancer cells than in normal cells. Whisker and box plot showing the distribution of correlation index (CI) for MCF10A (left) and MCF10CA (right) cells **(A)**. Plots were constructed using data from 11 FOVs from 2 experiments for each cell type, totalizing several hundreds of cells. The CI of each FOV was normalized to the median CI of normal cells for the same experiment. **: Wilcoxon rank-text p-value p=0.0151. Two examples of collective migration of live MCF10CA cells along trails are shown in **(B)**. Asterisks (*) indicate the hyaluronan-rich area that the migrating cell follows, and arrowheads show the trail that a moving cell leaves behind (left panel in B and **Supplementary Movie 2**). The right panel in B and **Supplementary Movie 2** show an example of leader-follower behavior, where the original position (0 min) of the leader cell is indicated by an arrowhead and the follower cell by an arrow. Red = hyaluronan and blue = nuclei.

We next interrogated the functional relationship between cancer-associated hyaluronan/EV-rich trails and the ability of cancer cells to coordinate their motion. To quantify coordinated cellular motion, we imaged MCF10A and MCF10CA cells overtime in an Incucyte Live-Cell Imaging System. For each field of view (FOV), we computed single-cell trajectories and defined a FOV-based correlation index (CI), that measures the degree of collinearity between the displacement directions of all pairs of cells across the FOV and all time-points (*Methods*). The CI defined a FOV-based global metric of collective cell motion. The median CI was 48% larger for FOVs showing cancer cells than for FOVs showing normal cells (**Figure 5A**, Wilcoxon rank-test p-value p=0.0151, N=11 FOVs per cell type). This increase in correlated motion index was not due to the limited number of FOVs (*Methods*). Therefore, collective cell motion is more coordinated in cancer cells than in normal cells (**Figure 5A**).

Then we sought to investigate correlations between coordinated motion and hyaluronan trails. We utilized time lapse live cell imaging combined with fHABC staining to monitor simultaneously single cell motion and the appearance of trails (selected time points: **Figure 5B**; full movies: **Supplementary Movies 1** and **2**). On the left panels of **Figure 5B**, a group of 4 cells that have produced a hyaluronan rich coating around them. Asterisk indicates a hyaluronan-rich area, which the migrating cell follows and reaches the area at 100 min time point. On the right, one leader cell is followed by a follower cell along the leader’s trajectory. These analyses strongly suggest that the follower cell tracks the hyaluronan-coated trail that the leader cell leaves behind.

### Hyaluronidase Digestion Decreases the Coordinated Migration Behavior of Tumor Cells

**Figure 6:**
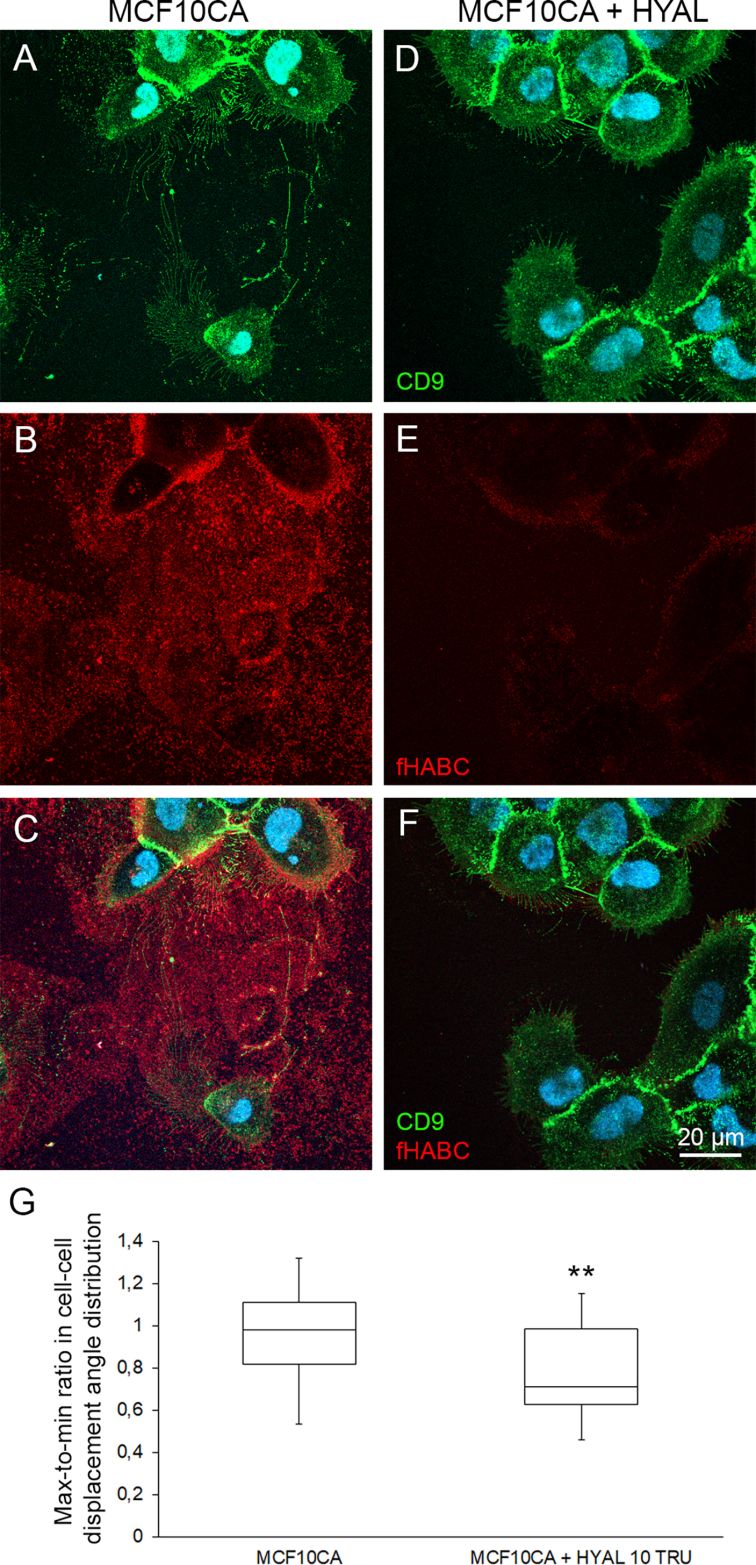
Pericellular hyaluronan promotes the coordination of cell motion in cancer cells. Comparison of live MCF10CA control cells **(A–C)** and hyaluronidase-digested cells **(D–F)** stained with FITC-labeled CD9 and fHABC. Blue = nuclei. Whisker and box plot showing the distribution of correlation index (CI) for untreated (left) and hyaluronan-degrading enzyme-treated (right) cancer cells **(G)**. Plots were constructed using data from 25 and 20 FOVs from 4 experiments for each condition respectively, totalizing several hundreds of cells. The CI for each FOV was normalized to the median CI of untreated cells for the same experiment. **: Wilcoxon rank-text p-value p=0.0068.

To test this hypothesis, we sought to investigate how the leader-followed behavior of MCF10CA cells is dependent on hyaluronan. In this purpose, we degraded the extracellular hyaluronan with the *Streptomyces* hyaluronidase, and compared MCF10CA motion coordination in presence and absence (control) of hyaluronan-digesting enzyme treatment of the extracellular medium. After 4 h digestion, most of the hyaluronan that in control untreated cells was abundant around CD9-positive plasma membrane protrusions and trails (**Figure 6B**) had disappeared (**Figure 6E**). Also, the plasma membrane protrusions and EVs decreased in hyaluronidase-treated cells (**Figure 6D**) as compared to control cells (**Figure 6A**). However, as previously shown (24), many of the lateral filopodia supported by the substratum were not dependent on hyaluronan coating (**Figures 6C, F**). Hence, hyaluronidase treatment effectively disrupted hyaluronan accumulation in cell trails.

Then, we repeated our time-lapse imaging experiments of moving cancer cells and quantification of the motion correlation in the presence of hyaluronidase-treatment to assess whether the coordinated motion of MCF10CA cells was dependent upon the presence of hyaluronan in trails. HYAL-treatment decreased the median CI by 28% (**Figure 6G**, Wilcoxon rank-text p-value p=0.0068, N=25 and 20 FOVs for untreated and treated cells respectively). Hence, extracellular hyaluronan promotes the coordination of cell motion in cancer cells.

### Cancer Cells Create Hyaluronan/EV-Rich Trails and Long Protrusions Also *In Ovo*

**Figure 7:**
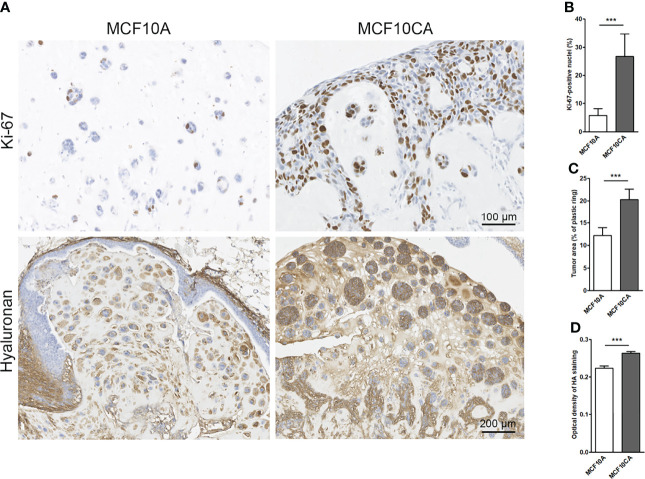
MCF10CA cells have a higher proliferation index, and they form bigger tumors with higher hyaluronan content than MCF10A cells in CAM model. Representative images of Ki-67-stainings (brown) and hyaluronan stainings (brown) of MCF10A and MCF10CA tumor sections **(A)** The proportion of Ki-67-positive nuclei in both tumor groups is shown in **(B)**, the average tumor area is shown in **(C)** and optical density of HA staining visualized with DAB in **(D)**; n = 8 in MCF10A and n = 10 in MCF10CA groups. **p < 0.001.

To mimic *in vivo* conditions, we cultured the MCF10 cells in chick chorioallantoic membrane (CAM) assays. Transplantation of both MCF10CA and MCF10A cells onto the CAM membrane of fertilized eggs resulted in tumor formation. The MCF10A cells formed small islands while MCF10CA tumors were formed by clearly bigger islands and chords (**Figure 7A**). As expected, MCF10CA cells showed a significantly higher proliferative index (**Figure 7B**) and formed significantly bigger tumors as compared to those of MCF10A cells (**Figure 7C**). Especially tumor cell-associated hyaluronan was high in MCF10CA tumors but was also increased in the stromal areas of the tumors formed by MCF10CA cells, while MCF10A cells had a less intense staining (**Figure 7A**). The hyaluronan staining of tumor sections was analyzed, and there was a significant increase in the optical density of staining in MCF10CA tumors as compared to MCF10A tumors (**Figure 7D**), which is in line with the results of the monolayer cell cultures.

**Figure 8:**
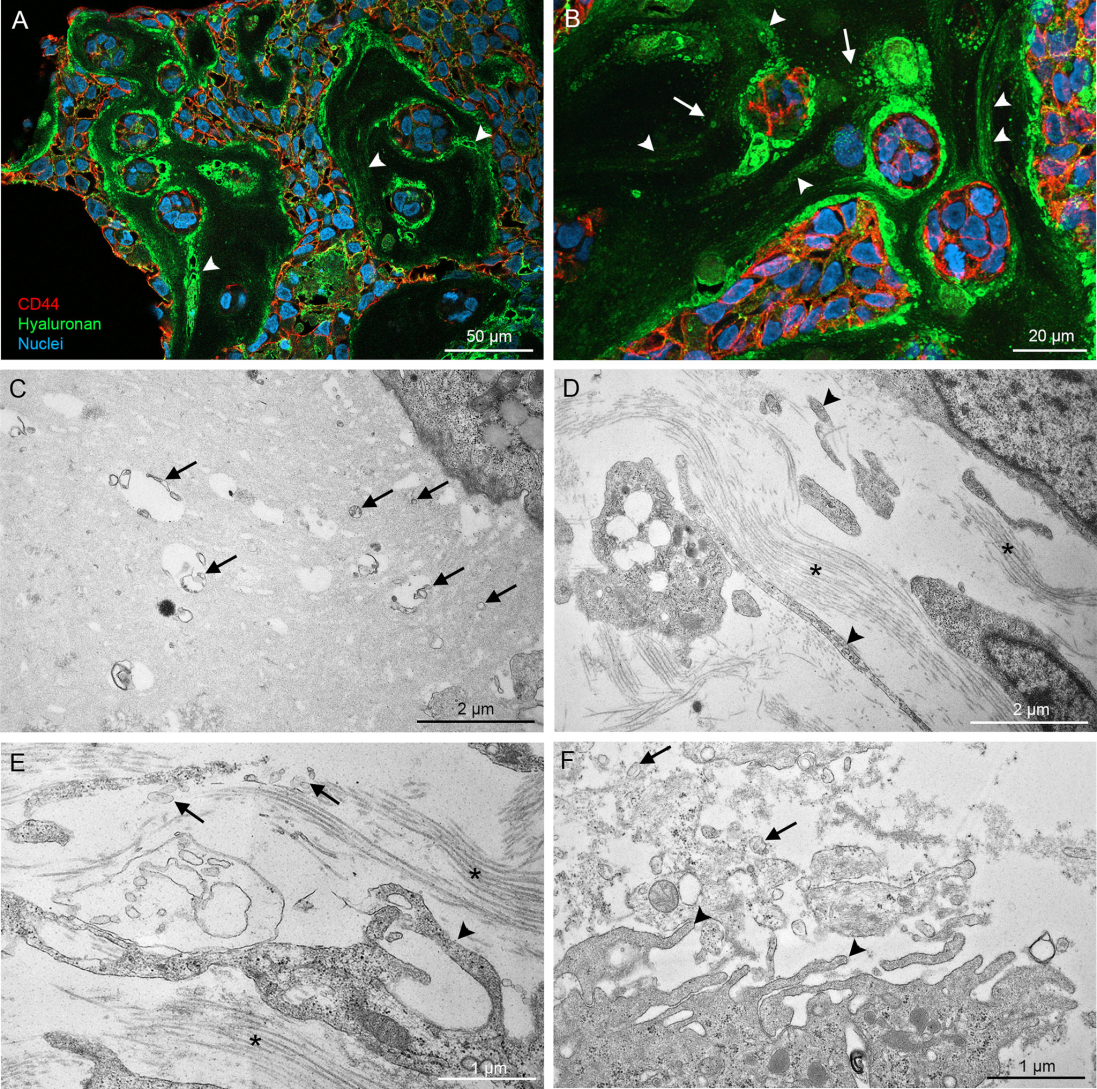
High resolution imaging of CAM tumor sections shows that hyaluronan-rich trails are formed in tumors *in ovo.* Confocal 3D projections from CAM tumor paraffin sections **(A, B)** derived from MCF10CA cells show high amount of hyaluronan between and around tumor cells and hyaluronan-rich trails (arrowheads in **A, B**). TEM images **(C–F)** show the numerous EVs of variable size and shape in the stromal areas (arrows in **C–F**). Asterisks (*) in **(D, E)** indicate collagen bundles.

CAM MCF10CA tumor paraffin sections were double stained with CD44 antibody and HABC for a more detailed analysis by confocal microscopy. Again, hyaluronan staining was very intense around and between tumor cells (**Figures 8A, B**), and hyaluronan-rich trails were detected in the stromal areas (arrowheads in **Figures 8A, B**), resembling the trails seen in monolayer cultures. Additionally, higher magnification revealed hyaluronan-positive particles (arrows in **Figure 8B**), which suggests the presence of hyaluronan-coated EVs. Because detection of thin plasma membrane protrusions and EVs in paraffin sections is challenging, transmission electron microscopy was utilized for more detailed morphological analysis of the cultures. Electron microscopy revealed EVs of variable size and morphology in the extracellular matrix (arrows in **Figures 8C–F**) and the very long plasma membrane protrusions (arrowheads in **Figures 8D, E**) that follow the orientation of bundles of collagen fibers (asterisks in **Figures 8D, E**). EVs were especially abundant in the proximity of plasma membrane protrusions pointing towards the stroma (**Figure 8F**). This data suggest that cancer cells also create hyaluronan/EV-rich trails to coordinate motion in 3D environments.

## Discussion

### The Leader-Follower Behavior of Cancer Cells Is Guided by Trails Left Behind by Leader Cells

In this study, we have discovered that aggressive MCF10CA breast cancer cells were forming trails coated with hyaluronan and EVs, and showed that “follower” cells stepped in those trails for coordinated, directional cell migration. The hyaluronan-coated trails discovered in this work resemble the EV containing “slime trails” introduced by Sung et al. (16), that are left behind migrating leader cells and promote the pathfinding behavior of follower cells in a paracrine manner (15). EVs contain multiple motility-promoting cargoes which might facilitate migration (15), although resolving how the effect of EVs is mediated would require more detailed investigation. It may be possible that the migrating cells internalize the EVs while they migrate over EV trails and use their motility-promoting cargoes to enhance their migratory capacity.

Cell-cell contacts and adhesion to the substratum or extracellular matrix are thought to be crucial for cell migration. This is particularly important during collective cell migration to maintain cells in contact with their neighbors while moving directionally (25, 26). Here we demonstrated a coordinated migration without direct cell-cell contacts, where single cells migrate in a coordinated way by following the ECM cues left by leader cells. Cellular adhesion to the ECM becomes particularly important for this kind of solitary migrating cells, including immune cells or metastatic tumor cells escaping the local tumor tissue.

Plasma membrane protrusions, especially filopodia are involved in substrate tethering and environment sensing of invasive tumor cells, and it is well known that migrating cancer cells assemble filopodia also in 3D environment (5). EVs are released from retraction fibers of migrating cells, as demonstrated using the pHluorin-CD63 EV marker (27). In agreement, we observed in this work very long filopodia or retraction fibers especially at the rear end of migrating breast cancer cells. From those filopodia, EVs were shedding as revealed by the CD9 marker, in line with the previous findings of filopodia as sources for EV shedding (28).

### Role of Hyaluronan in Coordinated Migration

The extracellular cues play a crucial role in paracrine interactions between the tumor cells and the ECM, either disabling or enabling tumor progression (4). These involve both mechanical and chemical migratory cues that regulate cell migratory behavior (25, 29). Hyaluronan expression in cancer is a predictor of poor prognosis (30), and its role in many processes related to cancer progression, including migration is well known (6). Hyaluronan secreted by leader cells can therefore provide in principle migratory cues to the follower cells.

Our results showed that breast tumor cells produced higher amounts of hyaluronan with higher average molecular weight than their normal counterparts, and that hyaluronan was positively affecting coordinated migration. In fibroblasts, it was shown that short hyaluronan chains stimulate migration (31), while in contrast high molecular weight hyaluronan in the ECM reduces migration of glioblastoma stem cells (32) and MDA-MB-231 breast cancer cells (33). Hence, it is possible that the effect of hyaluronan on migration is cell type/tissue-dependent. High expression levels of adhesion receptor CD44, which binds hyaluronan with high affinity, has been observed and shown to promote collective invasion in breast cancer (34, 35). This result could provide a mechanistic basis to explain the role of hyaluronan in coordinated migration. Interestingly, CD44- and integrin αVβ5-positive EVs released from retracting filopodia of trabecular meshwork cells were arranged in ‘trails’ (36). In addition to hyaluronan (37), tumor cells produce high amounts of mucins (38) on their cell surface, which creates a niche promoting tumor growth and survival. Our study does not exclude that other ECM proteins, possibly *via* interactions with cellular adhesion molecules including integrins, may play a role in facilitating the migration along these trails.

### EVs as Facilitators of Migration

Interestingly, hyaluronan synthesis is associated with enhanced EV shedding, which has been shown either by overexpressing hyaluronan synthases (8, 39, 40) or by removal of the glycocalyx by hyaluronidase digestion (41). Specific glycocalyx compositions such as mucin biopolymers and long-chain polysaccharides are secreted by tumor cells and drive formation of protrusions and secretion of EVs (41). We showed here that when cultured in identical conditions, aggressive MCF10CA cancer cells produce more EVs than their corresponding, close to normal breast epithelial cells. Although there is evidence that tumor cells produce more actively EVs compared with their nonmalignant counterparts (42, 43) some reports show no differences on the levels of plasma EVs between cancer patients and healthy people (44). The complexity of plasma samples with EVs from many cell types, as well as methodological pitfalls, may interfere with analysis of this data and obfuscate the differences between cancer and healthy patients. In addition, the secretion of EVs in cancer cells migration could also be cell type/tissue-dependent.

Many recent findings suggest that tumor EVs can prime premetastatic niches (45). Furthermore, EV secretion rate is enhanced in migrating cells as compared to non-moving cells and directional cell migration is dependent on EV secretion (16). There is recent evidence on EV shedding from retraction fibers as “footprints” (13), or “adhesive exosome trails” (27).

### Directional Tumor Cell Movements in 3D Environment

Fibroblasts are major producers of the ECM and often drive the collective migration of tumor cells through direct intercellular contact (46) and by protease- and force-mediated matrix remodeling (47). Typically, carcinoma cells move within tracks in the ECM behind the leading fibroblast (47). However, the results of this study support the findings (4) that in addition to cancer associated fibroblasts, cancer cells themselves may also produce and modify ECM to guide their own collective migration.

Mechanical properties of the tumor niche regulate tumor cell migration (32). ECM modeling and alignment are strongly correlated with promotion of cancer invasion, and cell invasion is oriented along collagen fibers, suggesting that alignment of collagen fibers relative to tumors regulates invasion (48). It has been reported that hyaluronan can alter the orientation of collagen network (32). Recent data suggest that increased hyaluronan concentration swells the size of collagen network pores, which are filled with hyaluronan molecules (33). This may be due to the high water binding capacity of hyaluronan, which increases the hydrostatic pressure to create space between collagen fibers. The ability of tumor cells to acquire a distinct “leader” cell phenotype may be triggered by these hyaluronan-rich “voids” formed between dense collagen networks. According to the data of our study, filopodia seemed to follow the orientation of collagen fibers. This suggests that hyaluronan, cellular protrusions and EVs *via* their molecular interactions such as MMP activity may create spaces between collagen fibers to facilitate or attract follower cell invasion. Interestingly, in a recent work, increased pressure of the ECM was shown to drive coordinated cellular motion, by a rapid burst-like streams of cervical cancer cells emerging in matrices with low collagen concentration (49). It is thus possible that also hyaluronan has a role in the regulation of pressure by creating areas of lower collagen concentrations to facilitate bursts of cancer cell invasion.

### Final Conclusions

This study showed that aggressive breast cancer cells migrate in a more coordinated way than normal cells. During migration, they leave trails containing EVs secreted from plasma membrane protrusions and hyaluronan, which guide the migration of follower cells. These trails tended to follow the direction of collagen fibers in 3D conditions. This study introduces a novel mechanism for hyaluronan as a guide for coordinated migration and supports the role EVs as facilitators of migration.

## Data Availability Statement

The raw data supporting the conclusions of this article will be made available by the authors, without undue reservation.

## Author Contributions

NA, SO, and KR contributed to conception and design of the study. NA, HK, ST, JC, JH, JM, SO, and KR performed the imaging, laboratory analyses and analysis of the results. NA, HK, ST, and SO performed the statistical analysis. KR wrote the first draft of the manuscript. NA, HK, and ST wrote sections of the manuscript. All authors contributed to manuscript revision, read, and approved the submitted version.

## Funding

This research was financially supported by Academy of Finland GeneCellNano Flagship (grant 337120), Jane and Aatos Erkko Foundation, Juselius Foundation and Mizutani Foundation.

## Conflict of Interest

The authors declare that the research was conducted in the absence of any commercial or financial relationships that could be construed as a potential conflict of interest.

## Publisher’s Note

All claims expressed in this article are solely those of the authors and do not necessarily represent those of their affiliated organizations, or those of the publisher, the editors and the reviewers. Any product that may be evaluated in this article, or claim that may be made by its manufacturer, is not guaranteed or endorsed by the publisher.

## Supplementary Material

Supplementary Figure 1 Schematics of the correlation index definition. **(A)** Exemplary FOV showing normal cell trajectories as output by TrackMate, at times Tk (top) and Tk+1 (bottom). Two exemplary cells labeled Ci and Cj are shown for illustrative purposes. The displacement vectors Di,k and Dj,k of the cells Ci and Cj respectively are shown as arrows on the bottom panel, together with the displacement angle θi-j,k made by the directions of these two displacements. Similar displacement angles were obtained for all pairs of cells and all time-points to obtain the distributions shown in **(B)**. **(B)** exemplary distributions of displacement angles for two FOVs representing normal cells (top) and cancer cells (bottom). The ranges of displacement values used to compute the peak occurrence (PO) and basal occurrence (BO), as well as the formula for the CI are indicated in red.

Supplementary Movie 1 An example video of trajectories of MCF10A cell cultures. The video was recorded for 12 h.

Supplementary Movie 2 An example video of trajectories of MCF10CA cell cultures. The video was recorded for 12 h.

Supplementary Movie 3 Time lapse movie of MCF10CA cells labeled with fHABC and NucBlue™. shows the coordinated movements of follower and leader cells along the hyaluronan-coated trails. Movie shows fHABC and NucBlue™ signals in MCF10CA cell culture during 100 min. The frames were collected in every 60 s and the movie plays at 7 frames/s.

Supplementary Movie 4 A time-lapse movie shows the coordinated movements of follower and leader cells along the hyaluronan-coated trails. Movie shows fHABC and NucBlue™ signals in MCF10CA cell culture during 60 min. The frames were collected in every 60 s, the movie plays at 7 frames/s and the total time is 60 minutes. The focus drift was adjusted a couple of times during the imaging.

## Abbreviations

CAM, chick chorioallantoic membrane assay; CLEM, correlative light and electron microscopy; ECM, extracellular matrix; EV, extracellular vesicle; HAS, hyaluronan synthase; NTA, nanoparticle tracking analysis; SEM, Scanning electron microscopy; TEM, transmission electron microscopy.
