# Supplementary material for: MCF10CA Breast Cancer Cells Utilize Hyaluronan-Coated EV-Rich Trails for Coordinated Migration
Source: Front Oncol. 2022 Apr 27;12:869417. doi: 10.3389/fonc.2022.869417 (PMC9091308; doi:10.3389/fonc.2022.869417)
Supplement: Supplementary Figure 1 — Schematics of the correlation index definition. (A) Exemplary FOV showing normal cell trajectories as output by TrackMate, at times Tk (top) and Tk+1 (bottom). Two exemplary cells labeled Ci and Cj are shown for illustrative purposes. The displacement vectors Di,k and Dj,k of the cells Ci and Cj respectively are shown as arrows on the bottom panel, together with the displacement angle θi-j,k made by the directions of these two displacements. Similar displacement angles were obtained for all pairs of cells and all time-points to obtain the distributions shown in (B). (B) exemplary distributions of displacement angles for two FOVs representing normal cells (top) and cancer cells (bottom). The ranges of displacement values used to compute the peak occurrence (PO) and basal occurrence (BO), as well as the formula for the CI are indicated in red. [file Presentation_1.pptx]

## Slide 1
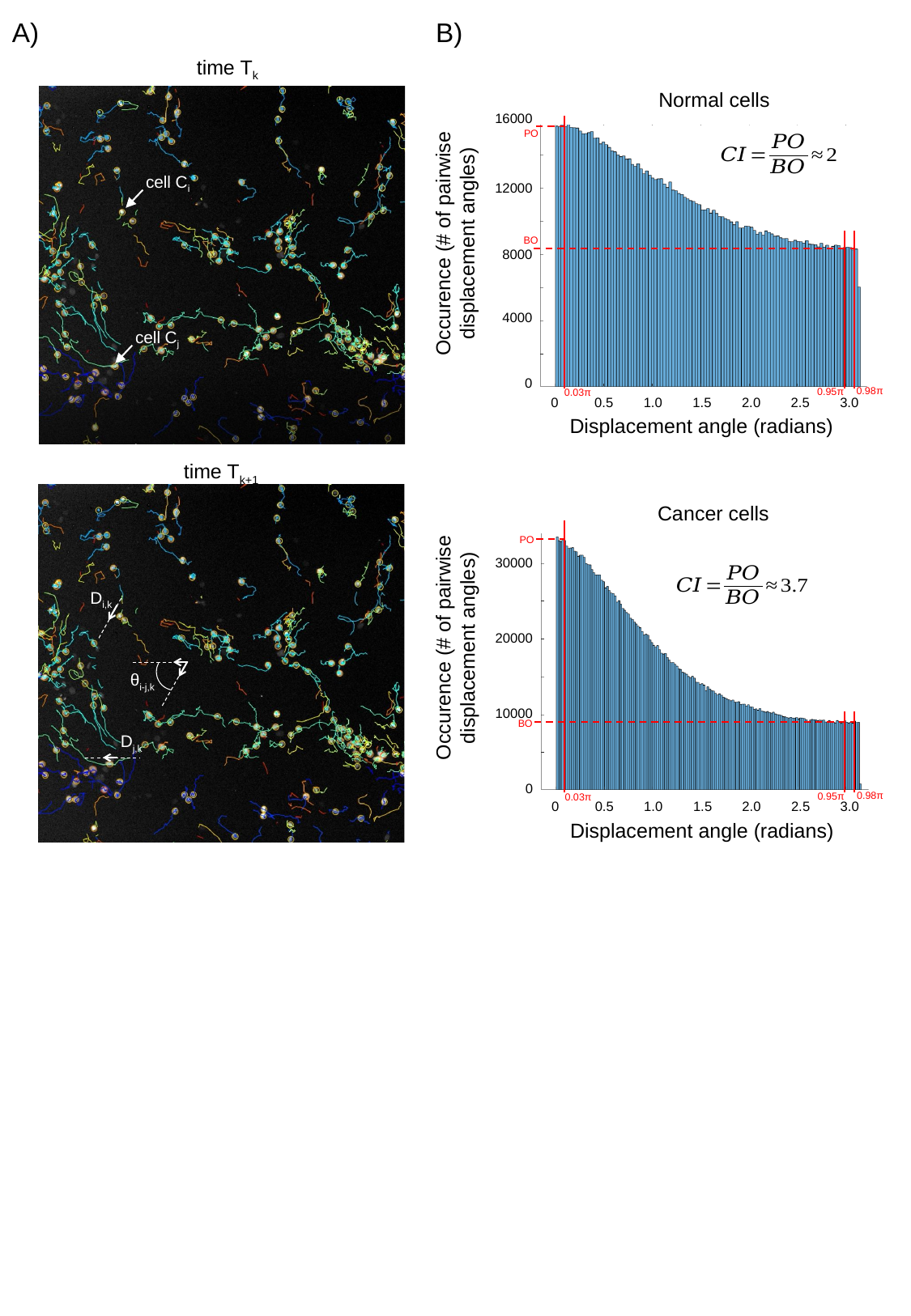

A)
B)
time Tk
16000
12000
8000
4000
0
PO
Occurence (# of pairwise displacement angles)
BO
0.98π
0.95π
0.03π
0
0.5
1.0
1.5
2.0
2.5
3.0
Displacement angle (radians)
Normal cells
cell Ci
cell Cj
time Tk+1
PO
30000
20000
10000
0
Occurence (# of pairwise displacement angles)
BO
0.98π
0.95π
0.03π
0
0.5
1.0
1.5
2.0
2.5
3.0
Displacement angle (radians)
Cancer cells
Di,k
θi-j,k
Dj,k
